# Four issues in undernutrition-related health impact modeling

**DOI:** 10.1186/1742-7622-10-9

**Published:** 2013-09-27

**Authors:** Noah Scovronick, Zaid Chalabi, Paul Wilkinson

**Affiliations:** 1 Department of Social and Environmental Health Research, London School of Hygiene & Tropical Medicine, 15-17 Tavistock Place, WC1H 9SH London, UK

**Keywords:** Nutrition, Malnutrition, Epidemiology, Health impact, Food prices, Modeling

## Abstract

Undernutrition modeling makes it possible to evaluate the potential impact of such events as a food-price shock or harvest failure on the prevalence and severity of undernutrition. There are, however, uncertainties in such modeling. In this paper we discuss four methodological issues pertinent to impact estimation: (1) the conventional emphasis on energy intake rather than dietary quality; (2) the importance of the *distribution* of nutrient intakes; (3) the timing of both the ‘food shock’ and when the response is assessed; and (4) catch-up growth and risk accumulation.

## Introduction

Undernutrition is estimated to account for 45% of all deaths in children under 5, and its reduction is a central component of the Millennium Development Goals (MDGs) [1,2]. As a result, health impact studies that evaluate the effects of disturbances, policies or interventions often attempt to quantify expected changes to undernutrition. Recent examples include analyses of climate scenarios, food price inflation and the global economic crisis [3-5].

In this paper, we highlight four methodological issues pertinent to undernutrition modeling and provide suggestions of how to approach them in future assessments. Our objectives are twofold: to improve the quality of undernutrition modeling, and to outline research areas in need of more empirical evidence.

No single paper could address all important issues relevant to undernutrition modeling. The four issues presented here were chosen for two reasons. First, they relate (directly or indirectly) to work on purchasing power (food prices and income). And second, each issue has benefitted from new and important epidemiological and economic research.

## Analysis

### Issue 1: dietary energy (calories) vs nutrient intake

Traditionally, most of the concern regarding food security and nutrition has centered on the availability of sufficient quantities of food rather than its quality [5]. The emphasis on energy is reflected in the broadly cited estimates of undernourishment provided by the FAO (Table 1), which forms the basis of MDG 1C. Food energy supply or undernourishment estimates are also the main (often the only) food-related inputs into other undernutrition models (Table 1). These energy-based models offer many valuable insights into past and future burdens of undernutrition, but do not portray all aspects of food insufficiency as they largely disregard micronutrient deficiencies. Excluding micronutrient-related diseases from analysis has several limitations.

**Table 1:** Common indicators of undernutrition, defined as an inadequacy of energy and/or other nutrients

**Indicator/model**	**Description**
Undernourishment	Estimates of ‘undernourishment’ are compiled annually by the Food and Agriculture Organization (FAO) for all developing countries. The estimates aim to identify the proportion of a population at risk of hunger, and therefore is more a measure of food security than health status.
	The FAO model assumes that the proportion of the population with mean energy consumption below a certain level is undernourished, and that the number below this threshold is estimable by assuming either a skew-normal or lognormal distribution of energy consumption defined by the population average and a coefficient of variation (FAO Statistics Division, [6]; FAO, [7]). Although commonly cited, experts have criticized this model for its overemphasis on calorie availability (as opposed to poverty) and its sensitivity to the input parameters, which are often difficult to measure (Svedberg, [8]).
	An important characteristic of the undernourishment model is that it is a population-based estimate. The measure is unfeasible for individual-level assessment; determining individual energy requirements would require long-term information on energy intake, physical activity and other factors such as pre-existing disease (e.g. diarrhea). Therefore, undernutrition at the level of the individual is normally determined using growth-based measures, and population surveys of growth faltering are preferable in determining the prevalence or incidence of undernutrition (see below).
Growth faltering	A common outcome of poor nutrition is faltered growth. Growth faltering is determined by comparing anthropometric measurements to international standards. Commonly used metrics of growth faltering include stunting (low height-for-age), wasting (low weight-for-height) and underweight (low weight-for-age). All three metrics are strongly associated with increased mortality from infectious disease and all-cause mortality [2,9].
	A model developed by Smith and Haddad [10] estimates the prevalence of underweight using a range of measurable population characteristics [10]. It derives from a regression equation whose parameters are: per capita food (energy) availability, the ratio of male-to-female life expectancy, female secondary education and access to safe water. Based on data from 1970-1996 from 63 countries, the model was designed to determine the main causes of past childhood undernutrition and has also been used to project future prevalence under different scenarios, including climate change [10,11]. A drawback of this model is the difficulty in projecting the parameters into the future.
	A newly developed model by Lloyd et al. [4] estimates the prevalence of stunting by combining estimates of undernourishment – using the FAO method – and a development score that is a function of GDP per capita and the Gini coefficient [4]. So far it has only been used to estimate the health impact from climate change [4].
Micronutrient deficiency	Micronutrient adequacy is measured in a variety of ways, including direct measurement (e.g. blood levels), analyzing dietary intake, or using the prevalence of related diseases [9]. Carriquiry [12] describes methods for assessing the prevalence of nutrient inadequacy in a group based on the distribution of intakes and requirements, but the models are not like those described above in that they do not relate specific risk factors to the prevalence of deficiency. To our knowledge no such model has been developed.
	Micronutrients of primary concern include zinc (associated with infectious disease and stunting), vitamin A (linked to blindness, childhood infections, and child mortality), iron (deficiency is the leading global cause of anaemia) and iodine (associated with thyroid function and cognitive development).
Dietary diversity	Dietary diversity – defined as the number of distinct foods consumed over a given reference period – can be measured using household survey methods. Dietary diversity has been associated with nutritional status in a range of settings, but is not itself a health outcome (it is an exposure) [13,14].
The Lives Saved Tool	The Lives Saved Tool was developed to estimate the potential impact on mortality of a range of different maternal and child health interventions [15]. Nutrition-related interventions include those affecting growth and/or micronutrient status. It has been used to estimate mortality impacts in a variety of settings [16].

The first is that the burden of disease from inadequate micronutrient intake may be considerable and should form an input into nutrition-related decision making – for example the specification of an intervention – alongside estimates of hunger or growth faltering; recent estimates indicate that vitamin A and zinc deficiencies each comprise around two percent of total child deaths, while other nutrient deficiencies also contribute substantial ill-health [2,9].

Second, the adverse health outcomes associated with growth faltering and micronutrient deficiencies are not independent. Too little zinc has been associated with increased stunting, even at mild to moderate levels of deficiency, and seemingly even when energy intake remains sufficient [17-19]. There is also suggestive evidence that other micronutrient deficiencies may constrain growth, at least at more severe levels of deficiency [18]. Increased susceptibility to infectious disease, characteristic of both growth faltering and micronutrient deficiencies, leads to feedback loops giving rise to what has been termed the “malnutrition-infection cycle” [20].

Therefore, models predicated entirely on one causal pathway – dietary energy in this case – may provide misleading estimates of the overall population health impact. Particular problems arise if a nutritional disturbance induces energy consumption to move in one direction and micronutrients in the other, or if energy and micronutrient intake move in a similar direction but are affected to substantially different degrees. In the former instance – when energy and growth-related micronutrients move in opposite directions – an energy-based model may mistakenly predict an improvement or deterioration of population-level nutrition status.

Instances where energy and micronutrient intake are affected differently (or to a substantially different extent) may not be particularly unusual. Econometric data from Mexico indicates that changes in household income has little impact on calorie (energy) intake but strongly affects the intake of many micronutrients (see below) [21]. Studies from Malawi, Guatemala and the United States show that when a suite of price changes occur, nutrient intake can be affected in virtually endless combinations and that individual price changes in certain food groups can result in the consumption of calories and zinc to move opposite directions, though substantial price changes would often be required for a meaningful effect [5,22,23].

Furthermore, several epidemiological studies have found that staple food consumption tends to remain relatively stable regardless of staple food price, but that higher proportional food expenditure on staples is positively associated with undernutrition (underweight and stunting) [24,25]. The authors suggest that the positive relationship results from reduced intake of nutrient-rich non-grain foods in the diet (lower dietary quality). However, reductions in energy may also contribute, as staples are not responsible for the totality of energy intake.

While the above discussion centers on undernutrition, the importance of differentiating between nutrients is well illustrated in a study from the United Kingdom that evaluated the potential health impact of a “fat tax” on unhealthy foods. Mytton et al. [26] added salt intake to a previous study that looked only at fat, concluding that the perceived beneficial effects on cardiovascular disease of reduced fat intake were likely to be offset by small increases in salt consumption [26].

A further, and related, issue is the possibility of double counting when summing disease burdens from multiple risk factors. Because some of the effects of micronutrient deficiencies are mediated through growth faltering, summing their associated health burdens, even when modeled appropriately, will yield possibly substantial overestimates. Black et al. [2,9] provide discussions of methods to calculate the population burden of disease from undernutrition from multiple risk factors [2,9].

The sometimes contrasting impacts on dietary energy and other nutrients is illustrated by income elasticity data from rural Mexico [21], which predicts the percent change in nutrient consumption in response to a one percent change in income.

The impact of a 15% increase in income, estimable by multiplying the reported elasticities by 15, suggests that calorie consumption responds little or not at all, vitamin A and C intakes increase markedly, and iron and zinc intakes decrease, although more modestly (Table 2).

**Table 2:** **Impact of a 15**% **increase in median income on intake (%) of select nutrients**

**Nutrient**	**Elasticity value‡**	**Intake change, %**
		**(15 x elasticity)**
Calories (energy)	−0.043†	−0.6
Vitamin A	1.244*	+18.7
Vitamin C	1.040*	+15.6
Iron	−0.378*	−5.7
Zinc	−0.184*	−2.8

‡From Skoufias et al. [21], estimated at the 50th percentile using quantile regression. We note that Skoufias et al. reported that elasticities differed depending on the population subgroup and the estimation technique. †Not significant (10% level). *Significant (1% level).

Thus, estimates of undernutrition derived only from calorie-based models would not predict a change in undernutrition levels despite the impacts on micronutrient status.

### Issue 2: determining if, when and how the shape of an intake distributions changes

All “cut-point” methods, whether to calculate undernourishment or deficiency of specific micronutrients, require data not only about the average intake in the population, but also about the distribution of intakes.

While knowledge about nutrient distributions in populations at (relative) equilibrium is often limited [8,12,27], how these distributions change after a major disturbance is even less well understood. Often, different segments of the population will be affected differently.

Interventions (e.g. food aid programs and some educational campaigns) sometimes explicitly target the most food insecure, while the urban poor may suffer more than the rural poor from food price inflation, because they rarely benefit from increased agriculture-related income [28]. Empirical data from economic studies have shown that households’ responsiveness to changes in food prices or income differ markedly when stratified by income [5,21].

Enhancing understanding of how distributions change in response to food disturbances is constrained by the substantial cost of food intake surveys; conducting a single survey can be prohibitively expensive for many countries, while sequential surveys over short time periods are nonexistent for all but the richest nations. Therefore, nutrition modeling often relies on shifting distributions based on changes to average intake.

A better understanding of distributional impacts not only allows for improved estimates of disease burdens, but can also inform the targeting of interventions.

To illustrate, we estimate the impact on vitamin A status of a hypothetical 15% decrease in income, again using data from the study of rural Mexican households considered above. The change in nutrient intake and associated deficiency is estimated using two methods. The first simulates the impact using a single elasticity calculated for the whole sample of households. For the second method, four elasticity values are used, estimated for the same sample but for each population quartile of expenditure (a proxy for income). All elasticities are applied to their corresponding, expenditure-stratified intake distributions, which were estimated from empirical data (see Additional file 1) [21].

The difference in baseline (and new) deficiency between the two methods results largely from imperfect information regarding the shape of the intake distribution (Table 3) (Additional file 1). Nevertheless, it is clear that Method 2 offers a much richer picture of the impact of a reduction in income. The vast majority of increased vitamin A deficiency occurs in the bottom two quartiles, a consequence of the relatively high elasticities and lower intakes (Table 3). Deficiency in the top two quartiles remains much more stable, despite still large estimated reductions in intake, indicating that these groups are fairly well insulated against an income shock, at least in terms of vitamin A status.

**Table 3:** Intake changes and percent deficient calculated using 1) a single population elasticity and 2) expenditure-stratified elasticities

	Baseline deficiency*, %	**Elasticity value**	**Change in income, %**	**Total change in intake, %**	New deficiency*, %	**Change in deficiency (percentage points)**
**Method 1**	**9.5**	**1.47†**	**−15**	**−22.1**	**15.0**	**+5.5**
**Method 2‡**	**13.8**	**n/a**	**−15**	**−23.8**	**20.0**	**+6.2**
*Quartile 1 (Bottom)*	*45.7*	*1.92*	*−15*	*−28.8*	*61.0*	*+15.3*
*Quartile 2*	*8.5*	*1.79*	*−15*	*−26.9*	*16.4*	*+7.9*
*Quartile 3*	*1.1*	*1.29*	*−15*	*−19.4*	*2.5*	*+1.4*
*Quartile 4 (Top)*	*0.1*	*1.35*	*−15*	*−20.2*	*0.2*	*+0.1*

*Deficiency was estimated using the fixed cut-point method, with the minimum requirement set at 37.5 μg of retinol equivalents. †All elasticities are from Skoufias et al. (2009) [21]. The authors presented multiple elasticities for the population based on different estimation techniques. This estimate was chosen because it is one preferred by the authors and is most coherent with the quartile estimates. ‡This row is the simple average of the four quartiles.

Similar to the above, Iannotti et al. [5] provide a good example of stratified responses to economic shocks in Guatemala, and the type of analyses that are possible when household-level data is available [5]. For example, they report that on average – looking at six nutrients and 14 food groups – consumption in the poorest income quintile was four times more responsive to income changes than in the richest quintile of households.

In addition to variation by income group, responses will likely also differ for other population strata, such as by age, rural/urban and ethnic background. Understanding the behavior of the segments of the population whose intakes are clustered near the minimum requirement has a particularly important bearing on the accuracy of impact estimates.

### Issue 3: the temporal pattern of disturbances and the subsequent response

In this section, we cover two related issues: how a given model represents the timing of a given disturbance – when it starts, when it finishes, and what happens in the interim – and how people respond to it over time.

Although short-term disturbances can have profound influences on individual- and population-level undernutrition [29-32], nutritional deficits often evolve more gradually and may be sustained. In either case, the timing of assessment may influence the estimates of impact. Shorter-term disturbances, like a drought or military conflict, tend to be assigned to a specific year (or years), although defining the start and end-points is often subjective. In other instances, as in analyses of future food prices, end-point projections often provide information about a future point in time (e.g. “wheat prices will be 25% higher in 2020”), but little on the trajectory of change up to that point.

A number of factors determine what people eat, including income, prices and preferences, amongst others [33-35]. Therefore, as one of these factors changes, for example through a food-price shock or an educational campaign, diets may change accordingly. The immediate dietary changes are sometimes predictable, but what is often more difficult to quantify is the persistence and pattern of the changes over time.

For instance, evidence from a variety of sectors show that when prices rise, consumers generally respond by buying less, but that the time-scale over which this occurs may vary; consumption may drop immediately, or over time, and in some cases may eventually revert towards the initial pattern [36-38]. Some economic modelers have noted that the assumption of an immediate, complete consumption response (a “jump”) rarely matches the “excess smoothness” seen in empirical analyses, and that models often behave better when incorporating phenomena such as “habit formation” [39,40].

A phased consumption response was recently demonstrated in the food sector in response to the dramatic price increases in high-fat, animal-sourced foods that followed the fall of the Soviet Union [37]. Longitudinal data show that fat consumption (as a proportion of energy consumption) dropped sharply after the initial price rise, but with the exception of the most expensive food items, returned to the baseline level after prices stabilized. The authors suggest that the pattern may have resulted from the Soviet regime’s prior promotion of fatty foods as well as from changing incomes.

Similarly, studies of behavioral adaptation indicate that households employ a range of coping strategies to absorb potentially adverse impacts on food security. For example, del Ninno et al. [41] reported that Bangladeshi households responded to the floods of 1998 with strategies such as modifying food production, changing employment patterns, and particularly by borrowing money or purchasing food on credit [41]. Authors have noted that different coping strategies tend to be implemented over different time-scales [41,42], beginning with attempts to limit exposure, followed by more drastic strategies such as liquidating assets and ultimately, migration [41].

Failure to account for both short-term and long-term consumer behavior may have notable implications for nutrition modeling; if a positive disturbance is a once-off event, like an educational campaign, assuming that the impact on diets will be sustained indefinitely may lead to an overestimate of potential benefits. Conversely, adverse impacts of negative disturbances, such as flood or famine, may be tempered by adaptation.

The issue of timing is illustrated by a recent assessment that used food-price elasticities to estimate the impacts on dietary energy intake and undernourishment resulting from the 37% increase in real food prices that occurred between 2006 and 2010 [3]. The analysis assumed linearity in the elasticities over the full range of the price inflation; in other words, a price increase from 1% to 2% caused an equivalent marginal impact on energy consumption as an increase from 36% to 37%. The authors thus estimated that the price inflation could have induced a 21% decrease in average energy consumption in Africa, potentially leading to ~200 million additional undernourished people.

The concern here is that the implied average energy intake is almost certainly unrealistic. A reduction in energy intake of 21% in Africa would cause average energy consumption to drop below the lowest level ever estimated by the FAO (the earliest estimates are for 1961). The estimate also contrasts with surveys of self-reported food insecurity conducted before and after the price shock, which found modest increases at most, although some of the disparity may have resulted from assumptions about income change [43].

The point is not to criticize the aforementioned study, and in fact the authors fully acknowledge the limitations in their method, choosing to interpret the estimates as “potential, perhaps maximum, risk” [3] p158S. But the analysis is a good illustration that not enough is known about responses to price and income shocks over time. It is especially difficult to estimate impacts when shocks are large, as they were in 2007/2008; elasticities are often calculated based on cross-sectional data and thus estimated using relatively small price variations.

Based on the available evidence, it is likely that consumption responses to large price increases are often non-linear (showing attenuated reductions in consumption beyond a certain point at which intakes are significantly reduced), and may change over time as individuals gradually find ways to compensate.

**Figure 1:**
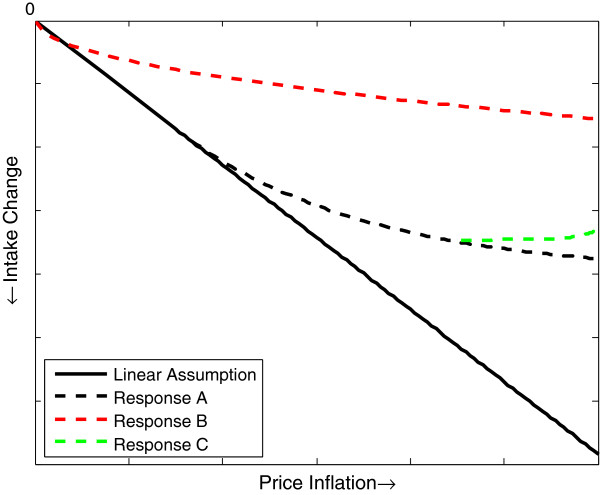
**Four potential responses to food price inflation.** See text for detailed explanation of each model.

Thus, responses may more realistically take one of the functional forms illustrated schematically in Figure 1. Response A assumes that the strength of the response (the elasticity) decays after a certain threshold of inflation, simulating, for example, a population that cannot endure further reduced energy consumption. After this biological limit is reached, more drastic measures (e.g. borrowing money, selling assets etc.) may be implemented to absorb the worsening shock. It may also be the case that an intake reduction only becomes perceptible after a certain point, even if it is not a health-relevant limit.

Similarly, in Response B, the elasticity also decays, but the decay begins immediately, representing a capacity (or need) to mitigate the price inflation from the shock’s onset. Shifting towards staple foods is one common coping strategy for minimizing intake reductions [3,24,25], and can be implemented quickly after a shock begins. The difference between Responses A and B may simply reflect different levels of poverty or food security; the pattern of the response is qualitatively similar.

Response C initially follows the curve in Response A, but then consumption recovers slightly. This may represent, for instance, the introduction of subsidies for home food production, cash transfers or the eventual distribution of food aid. The rebound is therefore a function of price only insofar as the continuously increasing prices trigger the (hypothetical) policy responses. A rebounding consumption response is more likely a function of time, as more time allows for more varied coping mechanisms [41].

Unfortunately, it is unusual to have all the information necessary to precisely define such curves. But there is often at least qualitative data on, for example, the frequency and size of meals consumed or about dietary diversity [3], and this knowledge could be the basis for outlining plausible responses. Testing different curves, even without full information, would be useful in characterizing possible health burdens when these uncertainties exist.

### Issue 4: the reversibility of undernutrition and accumulated risk

Epidemiological studies have demonstrated that children with suboptimal growth sometimes improve their nutrition status, though it appears easier to recover weight than height. The catch-up growth (weight or height) occasionally occurs spontaneously, without a change in environment, but can be profound if conditions improve substantially [44-47]. However, catch-up growth is not always straightforward; in addition to the amount of change in the environment, its speed and extent may depend on the age of the child, the severity of the initial growth faltering and age at puberty, amongst other factors [44-46]. Individuals classified as micronutrient deficient change status if intake becomes adequate, assuming the absence of non-nutritional contributors (e.g. hookworms).

If recovery from growth faltering or nutrient inadequacy occurs, it raises a question about risk reversibility, or the rate at which the previously increased risk of ill-health declines over time. This is sometimes referred to as a ‘cessation lag’. For instance, do children that improve their nutritional status from severely to moderately underweight transition immediately into the latter category in terms of the relative risk of death or disease? Or do the accumulated risks decrease more slowly? The same questions hold for micronutrient deficiencies. The converse is also important - whether increased risk after exposure occurs immediately or after some delay. (This is sometimes called the ‘onset lag’).

The literature on these issues is patchy and complex. A study from Brazil found that small-for-gestational-age (SGA) infants exhibiting catch-up growth (weight) had lower rates of hospital admissions and mortality than SGA infants that grew slower [47]. Hospital admission and mortality rates approached those of non-SGA children, but confidence intervals were wide, particularly for the latter.

There is also growing evidence that early undernutrition is associated with risk factors for chronic disease later in life, and that the association may be modified by catch-up growth [48]. For example, using a Finnish cohort, Eriksson et al. [49] demonstrated that males with the highest rates of death from coronary heart disease were thin at birth but had caught-up by age seven [49]. The seemingly reduced short-term, but enhanced long-term risks have led some experts to talk of a trade-off in the costs and benefits of catch-up growth [47,50].

In terms of cognitive and developmental outcomes, a study in the Philippines found that children severely stunted at age two but who had caught-up by age eight still had significant cognitive deficits compared to children who were never stunted [51]. There was some evidence that cognitive deficits decline with age. However, Crookston et al. [52] reported that Peruvian children that caught-up by age six did not differ from never-stunted children in tests of vocabulary and quantitative skills; persistently stunted children tested significantly lower [52]. Lozoff [53] found that Costa Rican children with iron anemia in infancy had worse motor and mental functioning at age five compared to other children, despite normal iron levels at the time of testing [53].

One possible explanation for the apparently contradictory results may be the presence of critical periods for reversing the effects of undernutrition. For example, improved schooling outcomes were associated with weight gain in children under two years of age, but not between two and four years [54]. Lozoff et al. [55], in a review of iron deficiency, suggests that “developmental windows of opportunity” may exist for only a limited time before the effects of iron deficiency become more permanent [55].

**Figure 2:**
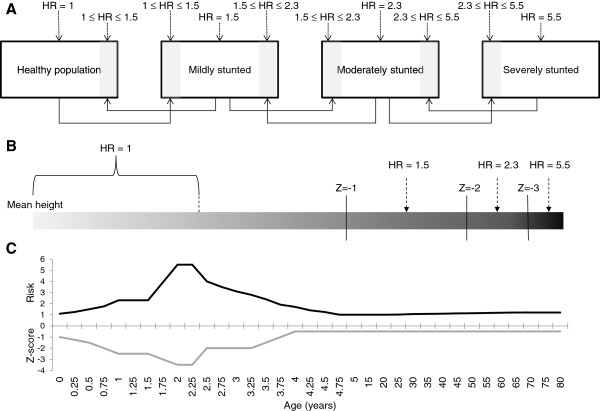
**Three possible models to represent catch-up growth and/or risk reversibility.** See text for detailed explanation of each model. Hazard ratios (HR) are based on Olofin et al. [56].

Despite these observed fluxes in nutrition status and, seemingly, in risk status, the traditional undernutrition paradigm allocates all individuals within an exposure category the same relative risk of death or disease. The alternative is to incorporate catch-up growth and/or risk decline estimates, where available, into more sophisticated analyses. We outline three possible modeling approaches, displayed diagrammatically in Figure 2, with varying degrees of complexity.

In the first diagram (Figure 2A), we show a dynamic conception of undernutrition that incorporates movement into and out of multiple categories of undernutrition. The diagram illustrates a situation where a proportion of the child population is classified as healthy or mildly, moderately or severely stunted. Children can move into or out of a given category if their growth status declines or improves from catch-up growth (or if they die). The key feature is that new entrants to a given category are assigned a mortality risk intermediate between the risk of the new group and that of the old group. Operational variables are stunting rates and rates of catch-up growth. Clearly, we have made a number of simplifications including the assumption that no child can move more than one nutrition category in a given time-step.

Although Figure 2A begins to account for catch-up growth and accumulated risk – the latter represented as time since arrival – it still treats individuals in each (sub) category as homogenous. In reality, there is a continuous flux of movement up and down the growth ladder. Figure 2B illustrates an alternative formulation, eliminating categories of stunting and instead conceiving of insufficient height as a continuum. Known mortality risks are reference points at specific locations on the continuum (here, the mid-point between Z-scores), with intermediate locations assumed to have intermediate risks that are a function of their distance from the nearest reference values. Movement to the right on the continuum represents increased stunting (and mortality risk), while movement to the left represents catch-up growth. A recent meta-analysis exploring the association of mortality with suboptimal growth while using uncommonly narrow Z-score bands supports the notion of a risk continuum, finding a monotonically increasing risk with decreasing Z-scores [56].

Despite its advantages, this model also has drawbacks. First, it requires estimates of growth velocities over time. Second, and more important here, the model does not truly incorporate any accumulated risks; for example, a newly stunted child with a Z-score of −2 would be assigned the same risk as a different child with a Z-score of −2 who previously had a lower Z score but caught-up. It does ensure that growth is a gradual process rather than a single (or series) of abrupt transitions.

The last model (Figure 2C) traces a single (hypothetical) child’s growth trajectory. The key feature of the diagram is that the mortality risk depends on whether the child is experiencing growth faltering or catch-up. During the growth faltering stage (age 0–2), the mortality risk tracks the Z-score without delay. During catch-up, the accumulated risk declines gradually. For example, the child has the same Z-score (−1.5) at age six months and 3.5 years, but the risk of the former is 1.5 while the latter is ~2.4 (values are hypothetical) – a result of accumulated risk. Furthermore, the child reaches normal height (Z ≥ −1) at four years of age and eventually experiences a period without increased risk of death until early adulthood when the risk again begins to gradually rise, representing the possibility of increased susceptibility to chronic disease.

The disadvantages of this last model are the relatively intensive data requirements and that algorithms for estimating risk decline and accumulation may become complicated if a child experiences multiple phases of growth faltering and catch-up; growth velocities are variable even in children with adequate nutrition. Nonetheless, it provides a more realistic formulation of what are likely dynamic features of growth patterns and risk.

## Conclusion and future directions

Our accumulating knowledge of the complex processes that lead to (and protect against) undernutrition suggests that overly simplistic models may yield inaccurate results when simulating the effects of food-related shocks, such as large changes in food price, on undernutrition.

One of the main benefits of engaging in health impact modeling is that the modeling process identifies research areas for which the evidence base is lacking. The four issues discussed in this paper imply a need for more empirical study and model development. Among the important questions that need to be addressed are:

1) What is the relative importance of different pathways (energy vs micronutrient) to growth faltering?

2) How do different disturbances influence the distribution (and not just the mean) of nutrient intakes in a population? How long do these sometimes short-term changes persist?

3) How do vulnerable populations adapt to disturbances that affect food security? How do short-term adaptive behaviors differ from longer-term strategies?

4) What factors inhibit or enhance catch-up growth? What is the trajectory of risk decline after catch-up growth? What are common lag times for the occurrence of chronic diseases associated with certain childhood growth patterns? 

The reduction of undernutrition is a key objective of international public health. Therefore, health impact modeling is an important tool in the evaluation of different policies and in understanding the consequences of different disturbances. Considering the high prevalence of exposure to undernutrition in many countries, and the associated mortality risks, even small changes in estimates have an important bearing on decision making. Therefore, increasing the quality of undernutrition modeling can have positive influences on public health and the achievement of related policy goals.

## Abbreviations

MDG: Millennium Development Goals; FAO: Food and Agriculture Organization; SGA: small-for-gestational-age.

## Competing interests

The authors declare that they have no competing interests.

## Authors’ contributions

NS conceived of and designed the study, performed model simulations and drafted the manuscript. ZC contributed to the model simulations and critically edited the manuscript. PW participated in the design of the study and critically edited the manuscript. All authors read and approved the final manuscript.

## Supplementary Material

Additional file 1 Method used for estimating vitamin A intake in the (expenditure-stratified) sub-populations. Click here for file
