# Supplementary material for: Four issues in undernutrition-related health impact modeling
Source: Emerg Themes Epidemiol. 2013 Sep 27;10:9. doi: 10.1186/1742-7622-10-9 (PMC3852289; doi:10.1186/1742-7622-10-9)
Supplement: Additional file 1 — Method used for estimating vitamin A intake in the (expenditure-stratified) sub-populations. [file 1742-7622-10-9-S1.docx]

**Additional file 1.** Method used for estimating vitamin A intake in the (expenditure-stratified) sub-populations

**Four issues in undernutrition-related health impact modeling**

Noah Scovronick^1^ , Zaid Chalabi^1^, and Paul Wilkinson^1^

^1^London School of Hygiene & Tropical Medicine, Department of Social and Environmental Health Research, 15-17 Tavistock Place, London WC1H 9SH, United Kingdom.

Phone: +44 (0)20 7927 2700, Fax: +44 (0)20 7927 2701

[Noah.Scovronick@lshtm.ac.uk](mailto:Noah.Scovronick@lshtm.ac.uk)

**Estimating nutrient (Vitamin A) intake for sub-populations**

Skoufias et al. (2009)[[1](#_ENREF_1)] report expenditure elasticities for different nutrients in rural Mexican households. They report overall elasticities for their whole sample, as well as expenditure-stratified elasticities. In order to explore the effect of a change in income (assuming expenditure is a good proxy), stratified elasticities must be applied to nutrient intake data for the corresponding sample. In addition to nutrient intake information for the whole sample, the authors report a mean, median and interquartile range (IQR) of nutrient intake for the first and fourth quartile of households, and also report that intake data was positively skewed in general.

Therefore, to apply the elasticities to intake data and calculate the percent deficient, we assumed a log-normal distribution of vitamin A intakes. For the whole sample and for the first and fourth quartiles, we determined the mean and standard deviation of the distribution using the median (which is also the geometric mean, GM, in a lognormal distribution) and the IQR, as follows:

Define $z$ = standard normal deviate such that $p(z>z_{p})=p$; so $z_{0.75}= -z_{0.25}=0.67$ and assume $x$ is a log-normally distributed random variable:

$x$~ lognormal(μ,σ)

where $\mu$ and $\sigma$ are the mean and standard deviation of

$$x_{p}=exp(z_{p}\sigma+\mu)$$

$$IQR= x_{0.75}-x_{0.25}=\exp\left( z_{0.75}\sigma+\mu\right)-\exp\left( z_{0.25}\sigma+\mu\right)= \exp\left( \mu\right)\left[ \exp{(z}_{0.75}\sigma\right)-\exp{(z}_{0.25}\sigma)]$$

$$IQR=GM ⨉ [exp({\sigma)}^{z_{0.75}}- exp({\sigma)}^{z_{0.25}}]$$

$$IQR/GM= exp({\sigma)}^{z_{0.75}}-exp({\sigma)}^{z_{0.25}}= exp({\sigma)}^{z_{0.75}}-exp({\sigma)}^{{-z}_{0.75}}$$

From here, we can re-write $IQR/GM$ =$c$, $\exp\left( \sigma\right)=a$ and $z_{0.75}=k$, giving:

$a^{k}- a^{-k}=c$

(1)

Therefore, if we allow:

$$y=a^{k}$$

(2)

Then we can substitute Equation (2) into (1), giving:

$$y-\frac{1}{y}=c$$

(3)

Equation (3) can be rewritten as:

$y^{2}-cy-1=0$

(4)

Which has the solution:

$$y=\frac{c\pm\sqrt{c^{2}+4}}{2}$$

(5)

Therefore from Equation (2):

$$a=\left( \frac{c\pm\sqrt{c^{2}+4}}{2} \right)^{1/k}$$

(6)

In equation (6) we can substitute 0.67 for k and IQR/GM for c, thereby solving for $a$, the geometric standard deviation (GSD).

For the second and third expenditure quartiles, for which no intake data was reported, we used linear interpolation on the medians and the logarithm of the geometric standard deviations from the first and fourth quartiles.

**References**

1. E. Skoufias, V Di Maro, T González-Cossío, Ramírez SR (2009) Nutrient consumption and household income in rural Mexico. Agr Econ 40: 657-675.
